# Subcellular Fractionation Analysis of the Extraction of Ubiquitinated Polytopic Membrane Substrate during ER-Associated Degradation

**DOI:** 10.1371/journal.pone.0148327

**Published:** 2016-02-05

**Authors:** Kunio Nakatsukasa, Takumi Kamura

**Affiliations:** Division of Biological Sciences, Graduate School of Science, Nagoya University, Furo-cho, Chikusa-ku, Nagoya, Aichi, Japan; Philipps-University Marburg, GERMANY

## Abstract

During ER-associated degradation (ERAD), misfolded polytopic membrane proteins are ubiquitinated and retrotranslocated to the cytosol for proteasomal degradation. However, our understanding as to how polytopic membrane proteins are extracted from the ER to the cytosol remains largely unclear. To better define the localization and physical properties of ubiquitinated polytopic membrane substrates *in vivo*, we performed subcellular fractionation analysis of Ste6*, a twelve transmembrane protein that is ubiquitinated primarily by Doa10 E3 ligase in yeast. Consistent with previous *in vitro* studies, ubiquitinated Ste6* was extracted from P20 (20,000 g pellet) fraction to S20 (20,000 g supernatant) fraction in a Cdc48/p97-dependent manner. Similarly, Ubx2p, which recruits Cdc48/p97 to the ER, facilitated the extraction of Ste6*. By contrast, lipid droplet formation, which was suggested to be dispensable for the degradation of Hrd1-substrates in yeast, was not required for the degradation of Ste6*. Intriguingly, we found that ubiquitinated Ste6* in the S20 fraction could be enriched by further centrifugation at 100,000 g. Although it is currently uncertain whether ubiquitinated Ste6* in P100 fraction is completely free from any lipids, membrane flotation analysis suggested the existence of two distinct populations of ubiquitinated Ste6* with different states of membrane association. Together, these results imply that ubiquitinated Ste6* may be sequestered into a putative quality control sub-structure by Cdc48/p97. Fractionation assays developed in the present study provide a means to further dissect the ill-defined post-ubiquitination step during ERAD of polytopic membrane substrates.

## Introduction

Endoplasmic reticulum (ER)-associated degradation (ERAD) is a conserved pathway that mediates the destruction of lumenal and integral transmembrane proteins. [1–6]. The importance of ERAD is underscored by the fact that human diseases including cystic fibrosis, heart disease, diabetes, rheumatoid arthritis, and neurodegenerative diseases can arise from the defects in the ERAD pathway [7, 8]. In *Saccharomyces cerevisiae*, two integral membrane E3 ligase complexes, the Hrd1 complex and the Doa10 complex, catalyze substrate ubiquitination during ERAD [1–3]. Hrd1 and Doa10 possess a RING domain on the cytosolic face of the ER. Current evidence suggests that degradation of substrates with a misfolded domain(s) in the lumen (ERAD-L substrate) is Hrd1 dependent [9, 10]. In addition, the degradation of integral membrane substrates that have misfolded domains in the transmembrane domain (ERAD-M substrate) is also Hrd1 dependent [10, 11]. In addition to ERAD-M type substrates, Hrd1 was proposed to recognize membrane substrates that persistently associate with the Sec61 translocon. This subclass of ERAD substrates has been termed ERAD-T (translocon-associated) [12]. By contrast, Doa10 mediates the turnover of integral membrane substrates with misfolded cytoplasmic domains (ERAD-C substrates) [9, 13]. Doa10 can also recognize intramembrane degron of a model tail-anchored protein [14]. Degradation of structurally complex proteins, particularly those that possess multiple spans, often depends on both Hrd1 and Doa10, likely because they have multiple misfolded domains [15, 16]. A recent study showed that quality control of proteins in the inner nuclear membrane, a specialized ER subdomain, depends on a third ER integral membrane E3 ligase complex that is composed of Asi1, Asi2, and Asi3 [17, 18]. These ER quality control E3 ligase complexes not only target misfolded proteins for elimination, but also contribute to the physiological regulation of metabolic enzymes [6, 19]. For example, HMG-CoA reductase (Hmg2 in yeast) [20, 21] and squalene monooxygenase (Erg1 in yeast) [22] are degraded through ERAD in a sterol intermediate-dependent manner. In addition, degradation of lanosterol 14-alpha-demethylase (Erg11 in yeast) is mediated by the Asi complex [17, 18].

Because the catalytic sites of the ubiquitination machinery and the proteasome are located in the cytosol, ER-lumenal soluble substrates must be transferred to the cytosol for ubiquitination and degradation by a process referred to as retrotranslocation or dislocation [23]. Single-spanning and polytopic membrane proteins, which contain exposed cytosolic segments, can access the E3 enzymes and the proteasome without extraction to the cytosol [24–26]. However, *in vivo* and *in vitro* evidence has suggested that both single-spanning and polytopic membrane substrates may be extracted to the cytosol before proteasomal degradation [27–34].

Retrotranslocation and extraction of ERAD substrates require the hexameric AAA-ATPase Cdc48 in yeast and p97 in mammals [35–37]. Although Cdc48 is a soluble enzyme, a portion of it is recruited to the ER membrane via Ubx2, an *UBA*/*UBX* domain-containing membrane protein [38, 39]. During retrotranslocation, a protein-conducting channel is thought to mediate the movement of substrates across the ER membrane. Although Hrd1 is currently considered as a channel candidate for ERAD-L substrates [23, 40, 41], the importance of the transmembrane domains of Hrd1 and Doa10 as channel components remains to be established, especially for polytopic membrane substrates [28]. In addition, lipid droplets formed at the ER membrane were proposed to facilitate the extraction and/or ubiquitination of ERAD substrates [32, 42, 43]. Nonetheless, the extraction of polytopic membrane proteins likely requires elaborate machineries because of their complexity and the difficulties associated with substrate solubilization in the cytosol before proteasomal degradation.

While further study is needed to fully understand the mechanisms by which polytopic membrane substrates are recognized and ubiquitinated by the E3 ligase enzymes during ERAD, much less is understood about how these substrates are processed at the post-ubiquitination step. To further analyze the retrotranslocation of ubiquitinated polytopic membrane substrates and to define their physical properties in the cytosol, we focused on a model ERAD-C substrate, Ste6*, an integral membrane protein with 12 transmembrane spans [27, 44, 45], and performed a series of subcellular fractionation assays. Based on a series of centrifugation analyses, we propose that ubiquitinated Ste6* could potentially be sequestered into a putative quality control sub-structure, which can be enriched in P100 fraction, by Cdc48/p97. The assay developed in the present study will be useful to further dissect the ill-defined post-ubiquitination step during ERAD of polytopic membrane substrates.

## Materials and Methods

### Strains and plasmids

Yeast strains used in this study were as follows: W303-1a (*MAT***a**, *can1-100*, *leu2-3*,*112*, *his3-11*,*15*, *trp1-1*, *ura3-1*, *ade2-1*), KNY140 (*MAT***a**, *can1-100*, *leu2-3*,*112*, *his3- 11*,*15*, *trp1-1*, *ura3-1*, *ade2-1*, *pdr5*::*HPH*, *pep4*::*LEU2*), KNY186 (KNY140 background, *ubx2*∆::*CgHIS3*), and KNY208 (KNY140 background, *cdc48-3*) were described in [46]. H1246 (*MATα*, *ADE2*, *can1-100*, *leu2-3*,*-112*, *his3- 11*,*-15*, *trp1-1*, *ura3-1*, are1*Δ*∷*HIS3*, are2*Δ*∷*LEU2*, dga1*Δ*∷*KanMX4*, lro1*Δ*∷*TRP1*) and its wild-type control strain SCY62 (*MAT***a**, *ADE2*, *can1-100*, *leu2-3*,*112*, *his3-11*,*15*, *trp1-1*, *ura3-1*) were generous gifts from Sten Stymne (Swedish University, Alnarp, Sweden) [47]. SM4460 (*MAT***a**, *his3*∆*1*, *leu2*∆*0*, *met15*∆*0*, *ura3*∆*0*) and SM5186 (*MAT***a**, *his3*∆*1*, *leu2*∆*0*, *met15*∆*0*, *ura3*∆*0*, *rad23*∆::*KanMX*, *dsk2*∆::*KanMX*) were generous gifts from Susan Michaelis (The Johns Hopkins University School of Medicine, Baltimore, MD). Plasmids used in this study were as follows: pSM1082 (2µm *URA3 Pste6-ste6-166*-*3HA*) encoding 3xHA tagged Ste6* (the tag is located at the first ER-lumenal loop) was a generous gift from Susan Michaelis. pKN66 (*CEN/ARS URA3 P*_*GAL1*_*-ste6-166*-*3HA-T*_*CYC1*_) was constructed by inserting a PCR-amplified open reading frame of *ste6*–*166*-*3HA* from pSM1082 into *Not*I-*Xho*I sites of pKN16, which is a pRS316-based plasmid with *GAL1* promoter and *CYC1* terminator [46].

### Assay for ERAD

Degradation of ERAD substrates was analyzed by cycloheximide chase as described previously [46, 48].

### Subcellular fractionation

Cells were grown to log-phase (OD_600_ = 0.5–1.5) at 30°C. Temperature sensitive strains were first grown at 25°C and shifted to 37°C for 1 h before cells were collected. Cells (20–30 OD_600_ equivalent) were broken either by vortexing in the presence of glass beads (method #1) or by extrusion through a polycarbonate filter (method #2). In the first method, cells were resuspended in lysis buffer [20 mM HEPES, pH 7.4, 50 mM KOAc, 2 mM EDTA, 0.1 M sorbitol, 1 mM DTT, 20μM MG132, 10 mM *N*-ethylmaleimide (NEM), and complete protease inhibitor cocktail (Roche)]. Glass beads were added and cells were disrupted by agitation on a Vortex mixer eight times for 30 sec with 30 sec intervals on ice between each cycle. The homogenate was collected and pooled after rinsing the beads with buffer 88, pH 7.4 (20 mM HEPES, pH 7.4, 150 mM KOAc, 250 mM sorbitol, and 5 mM MgOAc). In the second method, cells were converted to the spheroplast in lyticase buffer (0.7 M sorbitol, 75% YP, 0.5% glucose, and 10 mM Tris-Cl, pH 7.4) and lysed in buffer [50 mM Hepes-NaOH, pH 7.5, 200 mM sorbitol, 150 mM NaCl, 1 mM PMSF, and 10 mM 2-Mercaptoethanol (ME)] by extrusion through a polycarbonate filter with 3 μm pores (Millipore). In both methods, unbroken cells were removed by centrifugation at 300 g for 5 min at 4°C. Subsequently, the supernatant (S3) was centrifuged at 20,000 g for 20 min at 4°C. The membrane pellet was resuspended in buffer 88, pH 7.4. The resulting supernatant (S20) and the pellet (P20) fractions were further processed for western blotting or immunoprecipitation. Where indicated, S20 fraction was further centrifuged at 100,000 g using Type-50Ti rotor for 1 hour at 4°C to give P100 and S100 fractions. For western blotting, a portion of each fraction derived from the equal OD_600_ of cells was mixed with TCA at a final concentration of 10%, and proteins were precipitated by centrifugation at 20,000 g for 20 min at 4°C. The resulting pellet was rinsed with ice-cold acetone and solubilized in SDS-PAGE sample buffer. For immunoprecipitation, each fraction was solubilized in denaturing IP buffer (1× TBS, 1% SDS, 4 M Urea, 10 mM NEM, 10 mM EDTA, and complete protease inhibitor cocktail) by heating at 42°C for 30 min. Insoluble materials were removed by centrifugation at 15,000 g at room temperature. Subsequently, SDS and Urea were diluted ten-fold with dilution buffer (1× TBS, 2% TritonX-100, 10 mM EDTA, 0.5% sodium deoxycholate, 10 mM NEM, and complete protease inhibitor cocktail). This lysate was added to anti-HA antibody that was conjugated to Dynabeads Protein G (Invitrogen) and nutated overnight. The beads were washed four times with wash buffer (1× TBS, 2% TritonX-100, 0.1% SDS, 10 mM EDTA, 0.5% sodium deoxycholate, 10 mM NEM, and complete protease inhibitor cocktail). Proteins were eluted from the beads with SDS-PAGE sample buffer by heating at 42°C for 30 min.

### Sedimentation assay

Cells (approximately 30 OD_600_ equivalent) were broken with glass beads as described above, except that the disruption was performed in TED buffer [10 mM Tris-Cl, pH 7.6, 1 mM EDTA, 1 mM DTT, 1 mM PMSF, 10 mM NEM, and complete protease inhibitor cocktail (Roche)] containing 10% sucrose instead of lysis buffer. The homogenate was collected and pooled with rinses of the beads with the same buffer. After unbroken cells were removed by centrifugation at 300 g for 5 min at 4˚C, the supernatant (S3) was layered onto a 20–48% sucrose step gradient (420 μl of 20, 24, 28, 32, 36, 40, 44, and 48% sucrose in TED buffer). Centrifugation was performed at 27,300 rpm for 16 h at 4°C in a SW60Ti rotor (Beckman). Fifteen fractions (252 μl) were collected from the top. Samples were further processed as described in the subcellular fractionation section to detect ubiquitinated Ste6p* or other organelle marker proteins.

### Flotation assay

A flotation assay was performed essentially as described previously, with modifications [49, 50]. Cells were disrupted with glass beads in lysis buffer as described above, and S3 cell homogenate (supernatant from the 300 g spin) was prepared. This homogenate was loaded from bottom to top in a centrifuge tube as follows: 600 μl of 2.3 M sucrose (in flotation buffer containing 50 mM HEPES, pH 7.4, 150 mM NaCl, 5 mM EDTA, 1 mM DTT, 1 mM PMSF, and complete protease inhibitor cocktail), 170 μl of the S3 homogenate mixed with 630 μl of 2.3 M sucrose (in flotation buffer, the final sucrose concentration became approximately 1.8 M), 1.2 ml of 1.5 M sucrose (in flotation buffer), and 1.0 ml of 0.25 M sucrose (in flotation buffer). The tube was centrifuged at 27,300 rpm in a Beckman SW60Ti rotor for 16 h at 4°C. Aliquots (300 μl) were removed from the top to the bottom of the gradient. Samples were further processed as described in the subcellular fractionation section to detect ubiquitinated Ste6* or other organelle marker proteins.

### Antibodies and immunoblot analysis

Anti-HA and anti-Ubiquitin were purchased from Roche and BioMol, respectively. Anti-Sec61 antibody was a generous gift from Jeffrey L. Brodsky (University of Pittsburgh, Pittsburgh, PA). Anti-Kex2 and anti-Pma1 antibodies were purchased from Abcam. Anti-Pep12, anti-Dpm1, anti-Pho8, and anti-Pgk1 antibodies were purchased from Invitrogen. Immunoblots were incubated with the indicated primary antibodies and appropriate HRP-conjugated anti-mouse or anti-rabbit IgG secondary antibodies (Sigma). The HRP-chemiluminescent signal was visualized by enhanced chemiluminescence (Pierce, USA) or Luminata Forte Western HRP substrate (Millipore).

### Fluorescence microscopy

Yeast cells were incubated with 10 μg/ml of BODIPY 493/503 (Molecular Probes) for 30 min at room temperature. Stained cells were immediately analyzed by fluorescence microscopy using Axio Observer.Z1 (Carl Zeiss). Images were analyzed with Axio Vision 4.6.

## Results

### Ubiquitinated Ste6* is extracted to the cytosolic soluble fraction in a Cdc48-dependent manner *in vivo*

**Fig 1:**
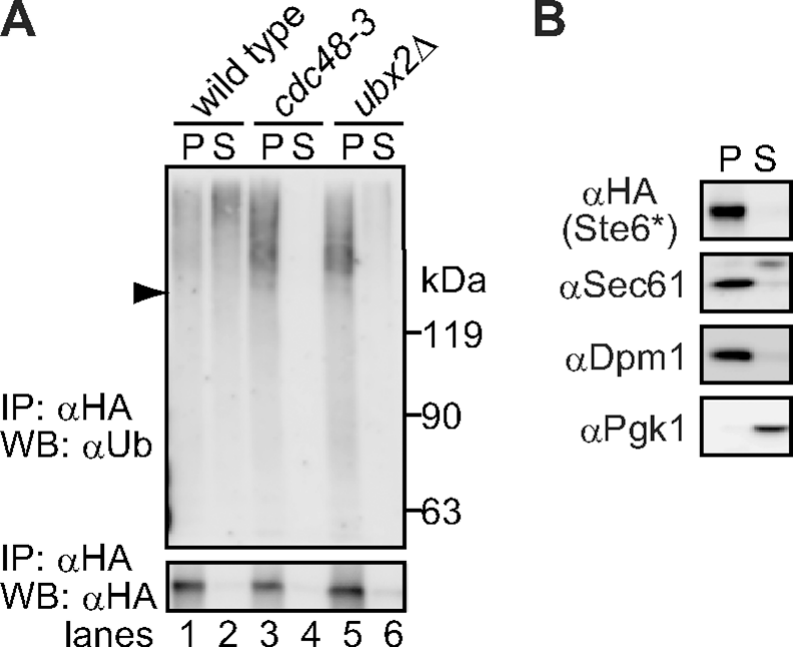
Subcellular fractionation assay reveals that the extraction of ubiquitinated Ste6* *in vivo* depends on Cdc48 and Ubx2. (A) Membrane (P20) and supernatant (S20) fractions were prepared from cells expressing Ste6*-3HA and subjected to immunoprecipitation under denaturing conditions with anti-HA antibody. Proteins were separated by SDS-PAGE and immunoblotted with anti-ubiquitin antibody or anti-HA antibody. Arrowhead indicates the position of unmodified Ste6*. (B) Membrane (P20) and supernatant (S20) fractions were prepared as above and analyzed with anti-HA (Ste6*), Sec61 (ER), Dpm1 (ER), and Pgk1 (cytosol) antibodies by western blotting.

Our previous *in vitro* study demonstrated that ubiquitinated Ste6* is extracted to the cytosol by Cdc48/p97 before degradation by the proteasome [27]. Hampton’s group also provided the *in vitro* evidence that full-length Hmg2, an ERAD-M substrate with seven transmembrane regions, is extracted to the cytosol by Cdc48 after ubiquitination [28]. To further analyze the extraction of ubiquitinated polytopic membrane substrate *in vivo*, we performed subcellular fractionation analysis. Cells expressing Ste6* were lysed and fractionated by centrifugation at 20,000 g. Subsequently, Ste6* was immunoprecipitated from each fraction under denaturing conditions and the distribution of ubiquitinated Ste6* was analyzed by western blotting. As shown in Fig 1A, unmodified Ste6* was exclusively detected in the pellet fraction (lane 1, bottom panel, approximately 90%). As a control, we confirmed that more than approximately 90% of the ER-membrane marker proteins including Sec61 and Dpm1 could also be recovered in the pellet fraction (Fig 1B). By contrast, ubiquitinated Ste6* was distributed in both pellet and supernatant fractions (Fig 1A, upper panel, lanes 1 and 2). When Cdc48 was inactivated by incubation of the temperature-sensitive mutant strain (*cdc48-3*) at a restrictive temperature, most of the ubiquitinated Ste6* was collected from the pellet fraction (lanes 3 and 4). This result was consistent with that of a previous *in vitro* analysis [27] and verifies the validity of the fractionation assay.

Based on previous results showing that at least a portion of Cdc48 is recruited to the ER membrane by Ubx2 for ERAD [38, 39], we examined the possibility that Ubx2 might facilitate the extraction of ubiquitinated Ste6*. As shown in Fig 1A, when the gene for *UBX2* was deleted, ubiquitinated Ste6* was predominantly detected in the pellet fraction, and only a faint amount of ubiquitinated Ste6* was collected from the supernatant fraction, indicating that Ubx2p is essential for the extraction of ubiquitinated Ste6* (lanes 5 and 6).

**Fig 2:**
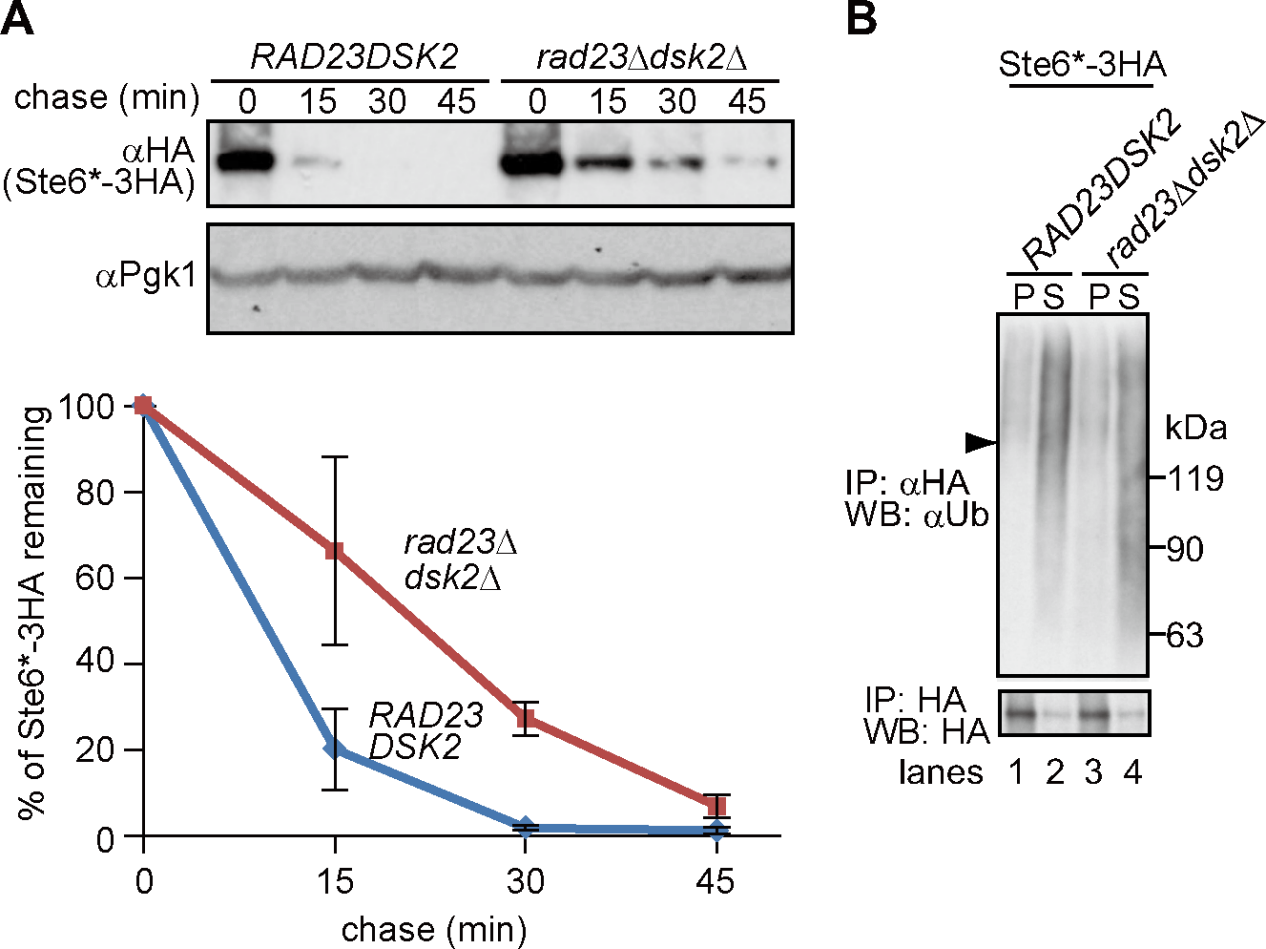
Rad23 and Dsk2 are dispensable for the extraction of ubiquitinated Ste6*. (A) Cycloheximide chase analysis of Ste6*-3HA was performed in *rad23*∆*dsk2*∆ cells. Quantification of three independent results was shown as a graph (error bars, S.D.). (B) Membrane (P20) and supernatant (S20) fractions were prepared from cells expressing Ste6*-3HA and processed as in Fig 1. Arrowhead indicates the position of unmodified Ste6*. Smeared band below the position of unmodified Ste6* may be due to a partial degradation of ubiquitinated Ste6* in *rad23*∆*dsk2*∆ cells (lane 4, upper panel).

We next asked if Rad23 and Dsk2, which deliver ubiquitinated substrates to the proteasome, could facilitate the extraction of ubiquitinated Ste6*. Rad23 and Dsk2 have been shown to be required for the degradation of CPY* (ERAD-L) and Hmg2 (ERAD-M) [51, 52]; however, to the best of our knowledge, their contribution to ERAD-C pathway has not been fully defined. We therefore performed a cycloheximide chase assay and found that the degradation of Ste6* in *rad23*∆*dsk2*∆ cells was moderately slowed (Fig 2A). However, the extraction of ubiquitinated Ste6* was unaffected by the deletion of *RAD23* and *DSK2* (Fig 2B), most likely because Rad23 and Dsk2 function after the Cdc48/p97-mediated extraction of Ste6*.

**Fig 3:**
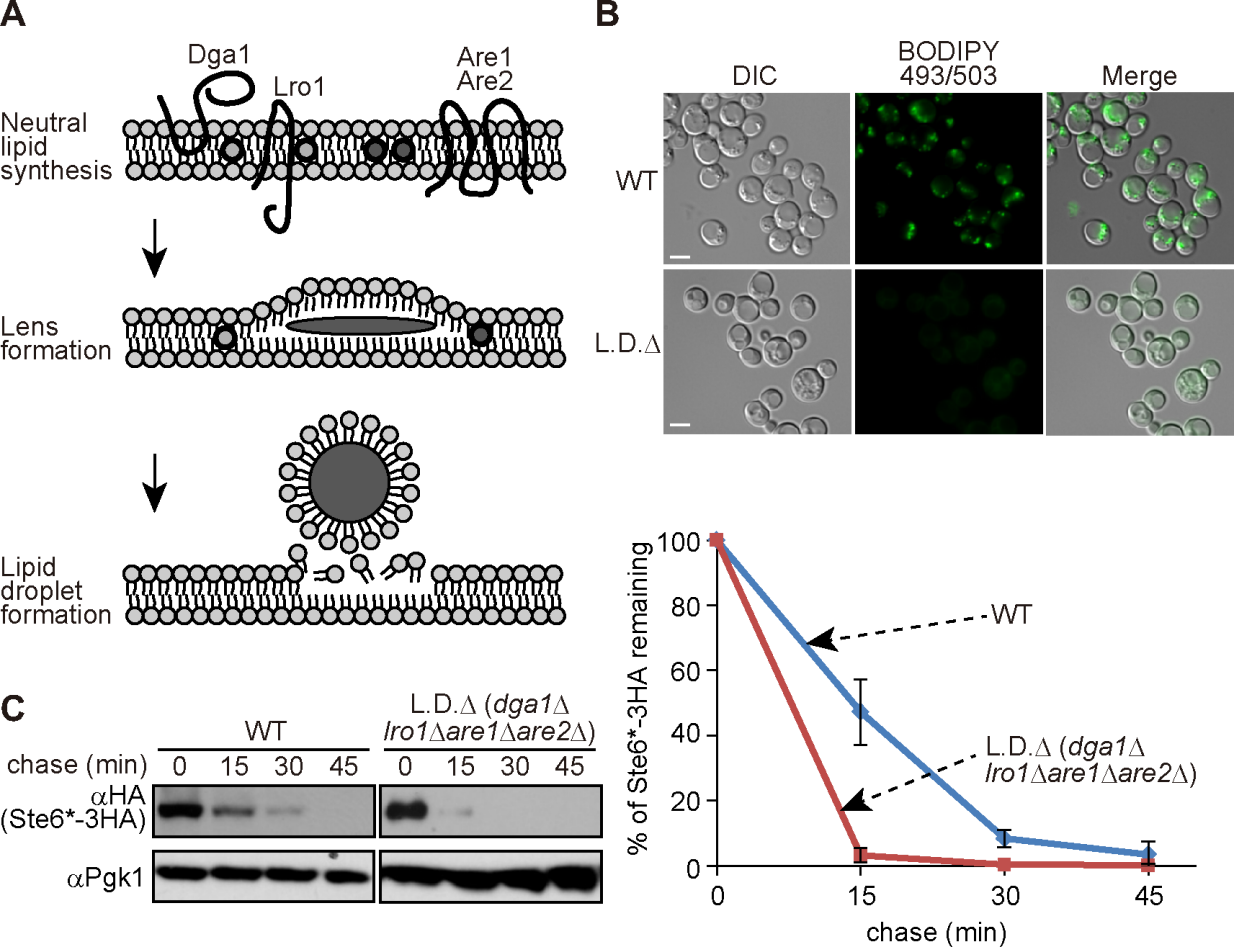
Lipid droplet formation is dispensable for the degradation of ERAD-C substrate. (A) Schematic representation of lipid droplet formation. (B) Fluorescence microscopic analysis of BODIPY 493/503-stained lipid droplets in wild-type and lipid droplet deficient quadruple mutant strains. (C) Cycloheximide chase analysis of Ste6*-3HA was performed in L.D.∆ (*dga1*∆*lro1*∆*are1*∆*are2*∆) cells. L.D.∆ or its isogenic wild-type cells were transformed with a plasmid encoding Ste6*-3HA under the control of the *GAL1* promoter. Cells were first cultured in synthetic complete media supplemented with 2% raffinose and shifted to YPGalactose media for 6 h to induce the expression of Ste6*-3HA. After translation was terminated with cycloheximide, cells were collected at the indicated time points and subjected to western blotting with anti-HA antibody. Because the expression of Ste6* was significantly lower in L.D.∆ cells than in wild-type cells, the blots for Ste6* in L.D.∆ cells were exposed longer than those for wild-type. Quantification of three independent experiments was shown as a graph (error bars, S.D.).

We next asked if whether a lipid droplet, which is derived from the outer leaflet of the ER ends up in the cytosol (Fig 3A), supports the extraction of hydrophobic transmembrane protein in yeast cells [42]. However, lipid droplet formation was previously shown to be dispensable for the degradation of CPY* (ERAD-L) and Pdr5* (ERAD-M) in yeast [53]. To determine whether lipid droplet formation was required for ERAD-C, we tested the degradation of Ste6* in lipid droplet deficient quadruple mutant cells (L.D.∆: *dga1*∆*lro1*∆*are1*∆*are2*∆) [47, 54–56]. The absence of lipid droplet in the quadruple mutant cells was confirmed by the immunofluorescence analysis using BODIPY 493/503 (Fig 3B). As shown in Fig 3C, the degradation of Ste6* in L.D.∆ cells was not inhibited, but was rather slightly accelerated. The present finding together with previous reports suggests that lipid droplet formation is not essential for the three major pathways of ERAD in yeast.

### Ubiquitinated Ste6* extracted from the ER membrane can be enriched in P100 fraction

**Fig 4:**
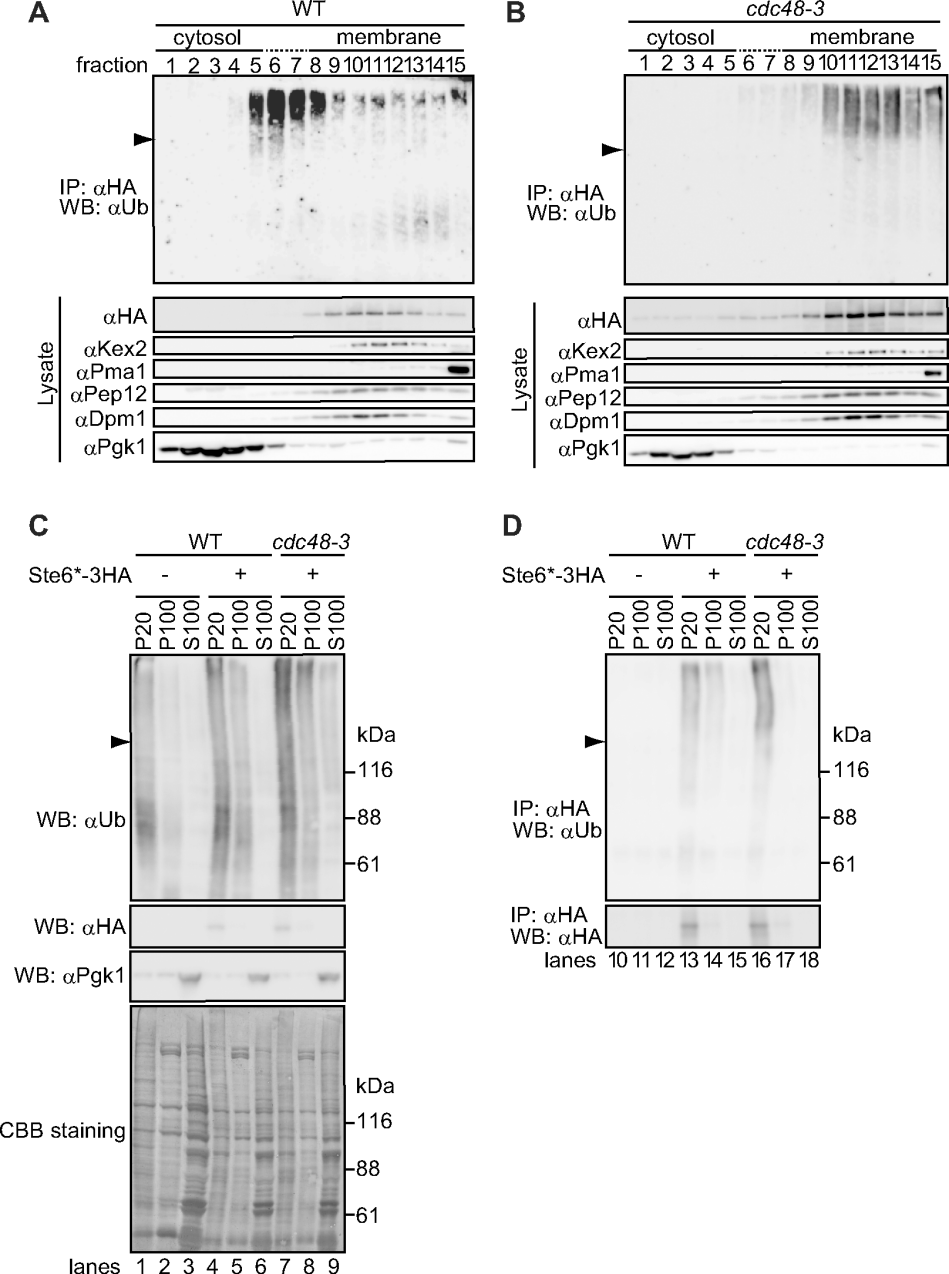
Ubiquitinated Ste6* is extracted from P20 fraction and sequestered into P100 fraction in a Cdc48-dependent manner. (A) Crude lysate (S3) prepared from wild-type cells was layered onto a 20–48% sucrose step gradient. After ultracentrifugation, 15 fractions were collected from top to bottom. Ubiquitinated Ste6* was immunoprecipitated from each fraction with anti-HA antibody under denaturing conditions, and its distribution was analyzed by western blotting with anti-ubiquitin antibody. The remaining aliquot from each fraction was analyzed by western blotting with anti-HA antibody (unmodified Ste6*) and with antibodies against organelle marker proteins (Kex2 (Golgi), Pma1 (plasma membrane), Pep12 (endosome), Dpm1 (ER), and Pgk1 (cytosol)). (B) Crude lysate (S3) was prepared from *cdc48-3* cells and analyzed as in (A). Arrowhead indicates the position of unmodified Ste6*. (C) Membrane (P20) and supernatant (S20) fractions were prepared from cells expressing Ste6*-3HA as shown in Fig 1A. The resulting S20 fraction was further subjected to the centrifugation at 100,000 g for 1 hour to give P100 and S100 fractions. The aliquot from each fraction was directly analyzed by western blotting with anti-ubiquitin antibody, anti-HA antibody (unmodified Ste6*), and anti-Pgk1 antibody. Note that less amount of ubiquitinated proteins were found in S100 fraction (lanes 3, 6, and 9) than in P20 fraction (lanes 1, 4, 7), although the amount of total proteins was higher in S100 fraction than in P20 fraction (see Coomassie Brilliant Blue R-250 (CBB)-stained membrane). Arrowhead indicates the position of unmodified Ste6*. (D) Ubiquitinated Ste6* was immunoprecipitated from each fraction prepared in (C) with anti-HA antibody under denaturing conditions and analyzed by western blotting with anti-ubiquitin antibody or anti-HA antibody.

To further analyze the subcellular localization of ubiquitinated Ste6p*, we performed sedimentation analysis. Cell extracts (S3) were prepared and layered onto a continuous sucrose density gradient. After ultracentrifugation, a total of 15 fractions were collected from top to bottom. A portion of each fraction was directly analyzed by SDS-PAGE, followed by western blotting with anti-HA to detect unmodified Ste6* and with antibodies against organelle marker proteins including Kex2 (Golgi), Pma1 (plasma membrane), Pep12 (endosome), Dpm1 (ER), and Pgk1 (cytosol). As shown in Fig 4A, Pgk1 was distributed in fractions 1–5, whereas Kex2, Pep12, and Dpm1 were distributed in fractions 8–15. Pma1 was predominantly collected from fraction 15, which is consistent with the notion that the plasma membrane is denser than other secretory organelles [57]. These results suggested that the present sedimentation protocol successfully separated cytosol and membranes derived from secretory organelles. We therefore designated materials in lanes 1–5 as cytosol fractions and those in lanes 8–15 as membrane fractions. We next tested the distribution of unmodified Ste6* and found that these species were predominantly collected from the membrane fractions (Fig 4A, αHA). By contrast, ubiquitinated Ste6* was distributed in fractions starting from 5 to heavier fractions, which partially overlapped with Pgk1 that was distributed in fractions 1–5. When Cdc48 was inactivated by using the temperature sensitive allele *cdc48-3*, ubiquitinated Ste6* was collected only from the membrane fractions, with no apparent signal detected in the cytosol fractions, whereas the distribution of unmodified Ste6* and other organelle marker proteins was unaffected (Fig 4B).

The simple pellet/supernatant fractionation assay at 20,000 g in Fig 1A suggested that a portion of ubiquitinated Ste6* was soluble like Pgk1. However, in Fig 4A, the distributions of Pgk1 and ubiquitinated Ste6* only partially overlapped. To investigate this difference, S20 fraction was further centrifuged at 100,000 g for 1 hour. In general, upon centrifugation of yeast lysate, >95% of ER membranes, mitochondria, and vacuolar membranes are precipitated at 13,000 g, while >95% of transport vesicles, ~40–90% of Golgi, and ~30–70% of endosomal membranes are precipitated at 100,000 g [58]. In addition, large protein complexes such as ribosomes and tubulin can also be precipitated under the same conditions. As shown in Fig 4D, ubiquitinated Ste6* was detected in P100 fraction, and only a faint amount was detected in S100 fraction (lanes 14 and 15). When Cdc48 was inactivated by using the temperature sensitive allele *cdc48-3*, ubiquitinated Ste6* was collected only from P20 fraction but not from P100 and S100 fractions, which is consistent with the result in Fig 1A. The cytosolic marker Pgk1 was recovered from S100 fraction (Fig 4C). The fact that ubiquitinated Ste6* is distributed in P20 and P100 fractions but not in S100 fraction may well explain our observation that ubiquitinated Ste6* was heavier than Pgk1 (Fig 4A). As a control, we performed the same experiment using lysate prepared from cells that did not express Ste6*-3HA and found that no apparent signal was detected in all fractions (Fig 4D, lanes 10–12), suggesting that the smeared signals were originated from Ste6*.

**Fig 5:**
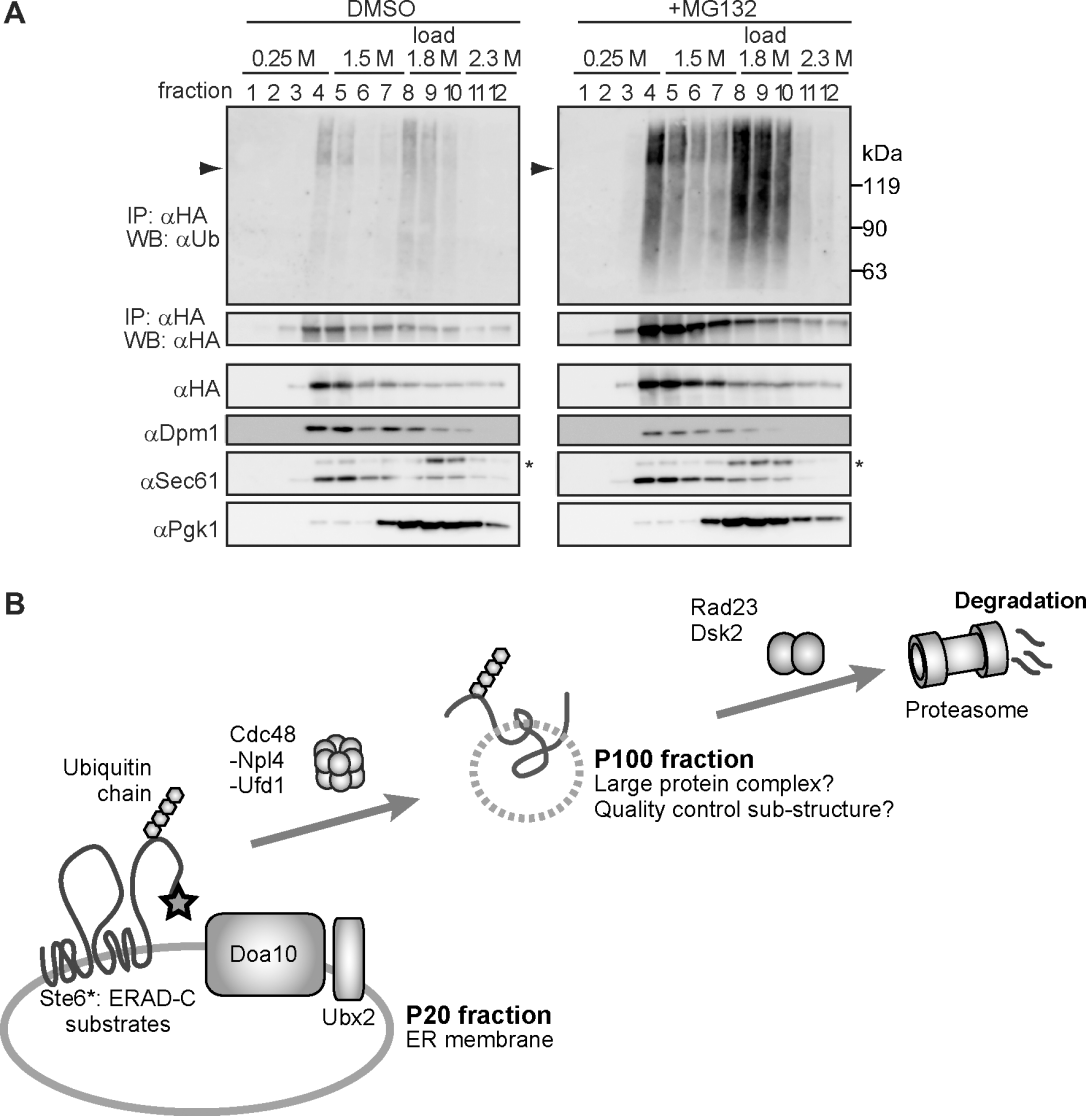
Flotation analysis of ubiquitinated Ste6*. (A) Crude lysate (S3) was prepared from cells expressing Ste6*-3HA and further separated using sucrose flotation gradient ultracentrifugation as described under “Materials and methods”. Aliquots were removed from the top to the bottom of the gradient. A portion of each fraction was directly analyzed by western-blotting with antibodies against organelle marker proteins. The remaining samples were subjected to the immunoprecipitation with anti-HA antibody under denaturing condition as in Fig 1A to detect ubiquitinated Ste6*. Asterisk indicates a non-specific protein(s) reacted with anti-Sec61 antibodies. Arrowhead indicates the position of unmodified Ste6*. Smeared band below the position of unmodified Ste6* may be due to a partial degradation of ubiquitinated Ste6* in cells treated with proteasome inhibitor MG132. (B) Model for the extraction of ubiquitinated Ste6* during ERAD. Ubiquitinated Ste6* is extracted from the ER membrane-enriched P20 fraction to P100 fraction in a Cdc48-dependent manner.

To further characterize the properties of ubiquitinated Ste6* *in vivo*, we performed a flotation analysis [49, 50]. Cell extracts (S3) were mixed with sucrose to a final concentration of 1.8 M and loaded near the bottom of a discontinuous sucrose gradient (see Materials and methods). The tube was ultracentrifuged at 100,000 g until reaching equilibrium. As shown in Fig 5A, membrane-associated proteins (e.g., Sec61 and Dpm1) floated up in the gradient to lower sucrose concentrations (fractions 4–7). By contrast, a soluble cytosolic protein (Pgk1) remained near the loading area (fractions 8–10), regardless of the presence or absence of the proteasome inhibitor MG132. Unmodified Ste6* floated up to lower sucrose concentrations with a single peak at fractions 4–7 in a similar manner to Sec61 and Dpm1. On the contrary, ubiquitinated Ste6* showed two distinct peaks: the first peak appeared at fractions 4–5, and the second peak appeared at fractions 8–10. The second peak almost completely overlapped with a cytosolic soluble protein, Pgk1. Given the technical limitations of biochemical fractionation assays, it would not be able to fully conclude that ubiquitinated Ste6* in lanes 8–10 is completely free from any lipids. However, because ubiquitinated Ste6* clearly showed two distinct peaks in this assay, the results strongly suggests that there are two distinct populations of ubiquitinated Ste6* with different states of membrane association.

## Discussion

The post-ubiquitination step during ERAD remains largely unclear, particularly for substrates that contain multiple transmembrane domains. In the present study, we performed subcellular fractionation analyses to determine the localization and physical properties of ubiquitinated Ste6* extracted to the cytosol during ERAD. We showed that ubiquitinated Ste6* is extracted from the ER membrane-enriched P20 fraction to S20 fraction in a Cdc48-dependent manner *in vivo*. This result was consistent with our previous *in vitro* observation. Intriguingly, ubiquitinated Ste6* in S20 fraction could be enriched by further centrifugation at 100,000 g. Although it is currently uncertain whether ubiquitinated Ste6* in P100 fraction is completely free from any lipids, membrane flotation analysis suggested the existence of two distinct populations of ubiquitinated Ste6* with different states of membrane association. Together, we propose that the P100 fraction containing ubiquitinated Ste6* could be used as a starting material to biochemically characterize a putative quality control sub-structure of misfolded polytopic membrane proteins (Fig 5B).

We and others previously demonstrated that ubiquitinated polytopic ERAD substrates are extracted to the cytosol by Cdc48/p97 before degradation by the proteasome [27, 28]. Similarly, HMGCoA-R in mammals was also shown to be extracted to the cytosol [32–34]. In addition, *in vitro* reconstitution analysis of the ERAD-L pathway in yeast suggested that Hrd1, which has six transmembrane regions, is autoubiquitinated and extracted from membrane by Cdc48/p97 [23]. These results support the idea that even polytopic membrane substrates can be extracted from the ER membrane to the cytosol during ERAD. However, we initially hypothesized that the extraction of transmembrane domains from the ER membrane is likely an energetically unfavorable reaction and might be a rate-limiting step, which could be slower than the proteasomal degradation in the cytosol. Therefore, the successful detection of ubiquitinated Ste6* in the cytosolic fraction *in vivo* was surprising. One possible explanation is that the downstream reactions mediated by the 19S proteasome (unfolding and/or de-ubiquitination of substrates) are rate-limiting steps, proceeding more slowly than the extraction. Furthermore, studies have suggested that the rate-limiting step during ERAD differs among substrate classes. For example, the degradation of certain ERAD substrates, such as the “Z” variant of the α-1 protease inhibitor (A1PiZ) and the cystic fibrosis transmembrane conductance regulator, was significantly slowed in cells lacking proteasome assembly chaperone Pba2 (also known as Add66), whereas others (carboxypeptidase Y* and pro-α factor) were not affected [59–63]. In addition, de-ubiquitination is a rate-limiting step in the degradation of certain proteasome substrates [64–66]. These observations suggest that the extraction from the ER membrane may be a rate-limiting step for some ERAD substrates, but it may not necessarily be the case for Ste6*.

It remains largely unclear how polytopic membrane substrates are extracted from the ER membrane to the cytosol. Currently, Hrd1 is a prime candidate for the protein-conducting channel that catalyzes retrotranslocation of lumenal substrates. However, one elegant study showed that the transmembrane domains of Hrd1 and Doa10 are dispensable for the extraction of a polytopic membrane substrate [28]. Moreover, degradation of Erg11, an ER-membrane protein, depends on the Asi complex, Ubc7, and Cdc48, but not on Hrd1 and Doa10 [17, 18]. It would be important to further analyze potential roles of the known quality control compartments including JUNQ (juxtanuclear quality control), IPOD (insoluble protein deposit), ERAC (ER-associated compartment), and (Q-bodies: dynamic inclusions containing misfolded proteins) [67–69] in the extraction of polytopic membrane substrates. In this regard, the P100 fraction in which ubiquitinated Ste6* is enriched could be used as a starting material for biochemical characterization of a putative quality control sub-structure in the cytosol during ERAD.

Recently, we found that the inactivation of Cdc48 triggers a formation of large complex containing 26S proteasome, ubiquitinated substrates, the Hrd1 complex, and the lumenal recognition factor, Yos9 [46]. Therefore, it is not unreasonable to speculate that Doa10 forms a similar transient complex with ubiquitinated Ste6* and the proteasome in a manner dependent on the Cdc48 ATPase cycle before the extraction step. Indeed, the proteasome can be immunoprecipitated with components of the Hrd1 and the Doa10 complex in an ATP-dependent manner from deoxy-BigChap-solubilized microsomes [70], although the presence of ubiquitinated substrates in this complex was not determined. Therefore, the 19S cap and Cdc48 are strong candidates for maintaining ubiquitinated Ste6* in the S20 fraction. Given a high solubility of ubiquitin molecule, polyubiquitin chain attached to the substrate could also serve a chaperoning role. In mammals, the Bag6 complex and the small glutamine-rich tetratricopeptide repeat-containing protein a (SGTA) were suggested to maintain the solubility of misfolded membrane proteins during ERAD [71]. Further analysis suggested that SGTA recognizes a non-canonical ubiquitin-like domain in the Bag6-Ubl4A-Trc35 complex to promote ERAD [72]. Moreover, both Bag6 and SGTA were shown to play a central role in dictating the fate of mislocalized membrane and secretory proteins [73–76]. Importantly, the yeast homolog of SGTA, Sgt2, interacts with Get5 (ortholog of Ubl4A), a protein involved in the insertion of tail-anchored proteins into the ER membrane [77–80]. Likewise, key components that mediate quality control of mislocalized membrane and secretory proteins also contribute to ERAD, although it is unclear whether Sgt2 plays a role in ERAD in yeast. Nonetheless, detection of such a highly transient interaction would require further technical development (i.e., spectrophotometric analysis or chemical cross-linking). Our fractionation assay would provide a means to further analyze the sequence of transient interactions that lead polytopic membrane substrates to the proteasome during ERAD.
